# Improving the Performance of Low-Carbon Ultra-High-Performance Concrete Through the Incorporation of Recycled Coarse Aggregate

**DOI:** 10.3390/ma19122621

**Published:** 2026-06-18

**Authors:** Yongquan Zhang, Xinyue Hao, Weimin Guo, Chengzhe Song, Fan Yang, Meiqi Cao

**Affiliations:** 1 Liaoning Transportation Planning and Design Institute Co., Ltd., Shenyang 110111, China; songzhe0323@163.com; 2 Transportation Institute, Inner Mongolia University, Hohhot 010021, China; zuohang201222@163.com (X.H.); yf18247929577@163.com (F.Y.); caomq890619@163.com (M.C.)

**Keywords:** ultra-high performance concrete, coarse aggregate, mechanical properties, volume stability, microstructure

## Abstract

**Highlights:**

- The 28-day compressive strength of UHPC can be increased by 23.32% through the inclusion of 15% steel slag aggregate with the size range of 5–10 mm.
- Both the autogenous shrinkage and drying shrinkage of UHPC can be reduced by the incorporation of coarse aggregate.
- The content of Ca(OH)_2_ decreased in UHPC matrix but increased in the ITZ of UHPC containing coarse aggregate.

**Abstract:**

Supplementary cementitious materials and aeolian sand have been used to produce low-carbon ultra-high-performance concrete (UHPC) due to their beneficial effects on the reduction in production cost and carbon emissions. However, low-carbon UHPC still faces some drawbacks, such as lowered mechanical properties, large shrinkage, and a tendency for cracking. This study proposed an approach to improve the performance of low-carbon UHPC by incorporating recycled coarse aggregate. The effects of recycled coarse aggregate type, particle size, and content on the workability and mechanical properties of low-carbon UHPC were investigated. Moreover, the internal relative humidity and volume stability of UHPC containing recycled coarse aggregate was also explored. At last, the hydration products and microstructure of UHPC was analyzed to shed light on the underlying mechanisms for the improved performance.

## 1. Introduction

Ultra-high-performance concrete (UHPC) is a new type of concrete material, which has superior mechanical strength, ductility, impact resistance, fatigue resistance and durability [1]. Compared to traditional concrete, the cement content in UHPC typically ranges from 900 to 1100 kg/m^3^, which is about twice that of traditional concrete [2]. However, only 39–48% of the admixed cement participate in hydration reactions in UHPC, while the remaining cement particles only serve as filler [3]. As it is well known, the cement industry accounts for 5–8% of the total anthropogenic carbon dioxide emissions [4]. In order to lower the carbon embodied during the production of UHPC, it is feasible to replace the portion of cement that not participate in the hydration reaction with supplementary cementitious materials (SCM), such as fly ash, ground granulated blast-furnace slag (GGBS), and silica fume [5]. The particle size of SCM is usually similar or finer than that of cement, which can not only play a role in filling but also participate in the pozzolanic reaction due to their latent reactivity [6].

When pursuing the improvement of concrete quality, UHPC typically employs fine aggregates with diameters not exceeding 2.5 mm while avoiding the inclusion of coarse aggregates [7,8,9]. Although quartz sand has become the preferred fine aggregate in UHPC due to its excellent properties, the process of its production entails substantial energy consumption, increasing the carbon emission associated with the manufacturing of UHPC. As a solution, aeolian sand [10], as a naturally formed siliceous material, has become a potential substitute for quartz sand in UHPC. Aeolian sand, characterized by its low moisture content, uniform particles and stable chemical composition, is mainly located in desert and Gobi areas. It has been demonstrated that aeolian sand can be used to prepare UHPC [11]. In this case, the environmental footprint of UHPC could be further reduced.

In this context, the low-carbon UHPC can be manufactured [12,13,14,15,16]. However, the shrinkage of low-carbon UHPC is still a concern that limits its application. Due to the employment of a reduced water-to-binder ratio, a high content of binder materials, and the absence of coarse aggregates, the shrinkage of UHPC is often larger than that of ordinary concrete [17]. The addition of coarse aggregate in UHPC can be a direct and effective approach to reduce the shrinkage of UHPC [18]. On one hand, the application of coarse aggregate can reduce the content of cementitious material required, resulting in a decrease in the content of cementitious matrix and thus a reduced shrinkage caused by cement hydration [19]. Meanwhile, coarse aggregate can play a certain anchoring role in the matrix, inhibiting the shrinkage of surrounding cementitious matrix [20].

At present, certain achievements have been made in the research on the shrinkage of conventional UHPC [21,22,23,24,25,26]. Various approaches have been proposed to mitigate UHPC shrinkage, including the incorporation of shrinkage-reducing admixtures, expansive agents, lightweight aggregates for internal curing, fiber reinforcement, and aggregate optimization [27]. Among these approaches, aggregate modification is considered particularly attractive because it can simultaneously improve dimension stability and reduce binder consumption. Recent studies have shown that recycled concrete aggregate (RCA) can be successfully incorporated into UHPC while maintaining satisfactory mechanical performance [28]. Due to the presence of adhered old mortar and relatively high water absorption capacity, RCA can act as an internal curing reservoir, gradually releasing absorbed water during hydration and thereby mitigating self-desiccation and autogenous shrinkage. Nevertheless, the effectiveness of this mechanism is highly dependent on aggregate quality, particle size, replacement level and pore structure.

In UHPC containing RCA, the evolution of the interfacial transition zone (ITZ) microstructure has been highlighted due to its importance in governing the mechanical and volumetric behavior of UHPC [29]. Meanwhile, sustainability-driven UHPC design has attracted increasing attention, focusing on reducing embodied carbon emission through the incorporation of industrial by-products, alternative sands and recycled aggregates [30,31]. Although previous studies have demonstrated that recycled aggregates can be incorporated into UHPC, most investigations focused on conventional UHPC mixtures with relatively high cement contents and quartz sand as fine aggregate. There still lacks the investigation on the influence of coarse aggregates on the volume stability of low-carbon UHPC [32]. In particular, no systematic comparison has been conducted regarding the effectiveness of steel slag coarse aggregate (SSCA) and recycled concrete coarse aggregate (RCCA) with different particle sizes and replacement levels in mitigating the shrinkage of low-carbon UHPC. The underlying mechanisms associated with hydration development and microstructure evolution also remain unclear.

In this study, low-carbon UHPC was prepared using high contents of fly ash, GGBS and silica fume as binder constituents, while aeolian sand was used as the fine aggregate. Two types of coarse aggregate, including SSCA and RCCA, were also incorporated into UHPC to reduce the shrinkage of UHPC. The effect of the types, particle sizes, and contents of coarse aggregate on mechanical properties and volume stability of UHPC were explored. At last, the hydration products and microstructure of UHPC containing coarse aggregate were investigated to elucidate the improvement mechanisms.

## 2. Materials and Methods

### 2.1. Materials

**Figure 1:**
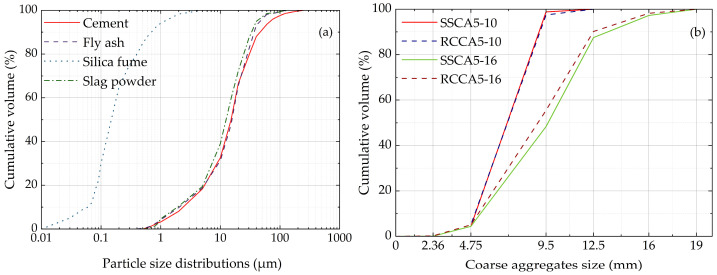
Particle size distributions of (**a**) binder and (**b**) coarse aggregate.

In this study, P·O 52.5 cement, fly ash, GGBS and silica fume were used as cementitious materials, purchased from Mengxi cenment company and Jurite building materials company (Hohhot, China), with their chemical compositions being listed in Table 1. The specific surface areas for these four types of binder materials were 380, 395, 487, and 19,500 m^2^/kg, respectively. Figure 1a shows the particle size distributions of cementitious materials. Aeolian sand with an apparent density of 2.65 g/cm^3^ and a fineness modulus of 1.27 was utilized as fine aggregate. The aeolian sand employed in this study was collected from desert regions in Chifeng, Inner Mongolia in northern China. The particle size distribution of aeolian sand is also present in Figure 1a.

**Figure 2:**
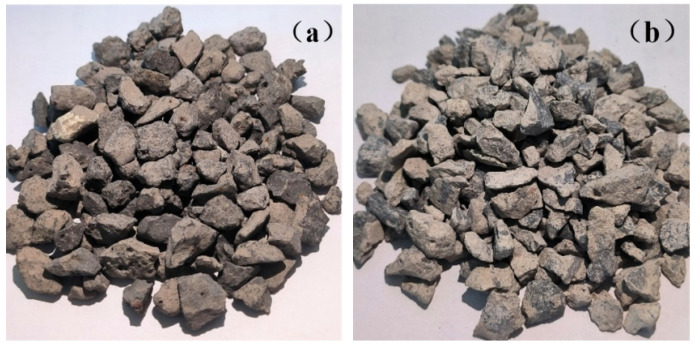
Appearance of coarse aggregate: (**a**) SSCA and (**b**) RCCA.

SSCA and RCCA, with particle sizes ranging from 5 to 10 mm and 5 to 16 mm, respectively, were utilized as coarse aggregate. Table 2 lists the physical properties of coarse aggregate. SSCA used in this study was obtained from a local steel manufacturing plant located in Baotou, Inner Mongolia and was processed through crushing and sieving to achieve the required particle size distribution. RCCA was produced by crushing waste concrete collected from demolished concrete structures, following by screening to remove impurities and obtain the desired grading. The particle size distribution of two types of coarse aggregate was present in Figure 1b. Figure 2 shows the appearance of coarse aggregates. A polycarboxylate superplasticizer with the water-reducing efficiency of 45% is employed to adjust the workability of the designed UHPC. A hook-shaped steel fiber with the length, diameter, and tensile strength of 13 mm, 0.2 mm and 2200 MPa, respectively, was used to improve the ductility of UHPC.

### 2.2. Mix Design and Sample Preparation

Nine series of UHPC mixture were prepared with the water-to-binder ratio kept at 0.16 and the volume dosage of steel fiber kept at 2.0%, with their mix design detailed in Table 3. The selection of water-to-binder ratio and steel fiber volume fraction were based on preliminary optimization experiments. In this case, they can provide satisfactory workability, fiber dispersion and mechanical performance. The control group (mixture Ref. in Table 3) was prepared without any coarse aggregate. The binder materials in the reference mixture included 360 kg/m^3^ of cement, 600 kg/m^3^ of fly ash, 120 kg/m^3^ of GGBS, and 120 kg/m^3^ of silica fume, while the content of fine aggregate was 1200 kg/m^3^ of aeolian sand. In order to examine the influence of coarse aggregate content on low-carbon UHPC, the replacement level of coarse aggregate was set as 5%, 10%, 15%, and 20% by volume of the UHPC matrix. It should be noted that these four replacement levels were selected based on the experimental results, which exhibited notable influence on the mechanical properties and volume stability of UHPC. In addition, two size ranges, namely 5–10 mm and 5–16 mm, were applied for each coarse aggregate replacement level, as detailed in Table 3. The 5–10 mm fraction represented relatively smaller coarse aggregates, while the 5–16 mm fraction provided a larger maximum aggregate size and potentially stronger shrinkage restraint. By comparing these two gradations, the effect of aggregate size on volume stability and microstructural development can be systematically evaluated.

Compared with conventional UHPC mixtures reported in the literature, which typically contain about 900 kg/m^3^ cement and 1000 kg/m^3^ quartz sand [33], the proposed mixture reduced cement consumption by approximately 60%. A simplified embodied carbon estimation was further conducted using typical emission factor from the literature (0.85 kg CO_2_/kg for cement, 0.02 kg CO_2_/kg for fly ash, 0.03 kg CO_2_/kg for silica fume, 0.005 kg CO_2_/kg for aeolian sand, and 0.01 kg CO_2_/kg for quartz sand) [34]. The estimated CO_2_ emission of the proposed UHPC was approximately 328 kg CO_2_/m^3^, whereas a conventional UHPC containing 900 kg/m^3^ cement and 1000 kg/m^3^ quartz sand would generate approximately 775 kg CO_2_/m^3^. This corresponded to an estimated reduction of about 58% in CO_2_ emission. In this case, the term “low-carbon UHPC” referred to the substantial reduction in cement consumption and associated embodied carbon emissions, rather than a complete life-cycle assessment.

During the mixing, cement, fly ash, GGBS, silica fume, aeolian sand with or without coarse aggregate were first dry-mixed for 4 min using a mixer at a rotational speed of 60 rpm. Subsequently, water and superplasticizer were added and stirred for another 5 min at 120 rpm. At last, steel fiber was gradually introduced and mixed for an additional 5 min at 120 rpm. The fresh UHPC mixture was cast into mold and covered with plastic film for 24 h. All mixing and casting procedures were conducted in a laboratory environment at a temperature of 20 ± 2 °C and a relative humidity of 60 ± 5%. After demolding, UHPC samples were transferred to curing room maintained at 20 ± 2 °C and a relative humidity higher than 95% until reaching the specific ages.

### 2.3. Testing Methods

#### 2.3.1. Workability

After finishing the mixing, the slump flow of the UHPC mixtures was evaluated in accordance with the Chinese standard GB/T 50080-2016 [35]. The average value of two diameters perpendicular to each other was recorded as the fluidity of UHPC. For each mixture, the slump flow was measured twice and the average value was reported.

#### 2.3.2. Compressive Strength

The compressive strength was evaluated according to the Chinese standard GB/T 50081-2019 [36] using UHPC sample with dimension of 100 × 100 × 100 mm. The test was conducted at the curing time of 7, 28, 90 and 180 days, respectively. During the testing, the loading rate was kept at 1.0 MPa/s. Three specimens were tested for each mixture and curing age. The reported compressive strength corresponded to the average value, while the standard deviation was presented as an indicator of experimental variability.

#### 2.3.3. Internal Relative Humidity

The internal relative humidity of the UHPC samples was measured using a hygrometer with an accuracy of ±0.3%. Prior to testing, the sensor was checked according to the manufacture’s calibration specifications. During the casting of UHPC mixture into mold, the hygrometer was put at the geometric center of the sample using a PVC pipe to ensure consistent sensor location among all samples. After installation, the opening around the sensor and PVC pipe was sealed to prevent moisture exchange between the UHPC sample and the external environment. Following the hardening of UHPC, the relative humidity inside the UHPC sample was continuously monitored for 7 days [17]. The PVC pipe served only as a positioning aid and occupied a negligible volume relative to the sample size. In this case, the influence of PVC pipe on the internal humidity evolution was considered minimal.

#### 2.3.4. Autogenous Shrinkage

As soon as the mixing procedure was completed, the fresh UHPC mixture was casted into prism molds (515 × 100 × 100 mm) installed in the NELD-ES730 autogenous shrinkage testing system (Beijing NELD Intelligent Technology Co., Ltd., located in Beijing, China). The UHPC samples were sealed by three layers of plastic films to prevent external moisture exchange. Two laser displacement sensors were positioned at both ends of each sample to continuously monitor length changes. The autogenous shrinkage measurement started immediately after casting and the deformation data were automatically recorded at 15 min intervals for 72 h. All measurements were performed in a laboratory environment maintained at 20 ± 2 °C. Prior to testing, the laser sensors were calibrated according to the manufacturer’s instructions to ensure measurement accuracy and stability.

#### 2.3.5. Drying Shrinkage

After finishing the autogenous shrinkage test, UHPC samples were demolded from the autogenous shrinkage testing instrument and transferred to the drying shrinkage chamber, in which the temperature and relative humidity were kept at 20 ± 1 °C and 50 ± 5%, respectively. The drying shrinkage test was conducted in accordance with the Chinese standard GB/T 50082-2024 [37]. The length of UHPC sample was measured continuously until the drying shrinkage gradually stabilized.

#### 2.3.6. Shrinkage Prediction Models

Four commonly used shrinkage prediction models, including the CEB-FIP model [38], B3 model [39], NF P18-710 model [40] and DEB-FIP model [41], were employed to evaluate the applicability of existing prediction approached to UHPC. Among these models, DBJ43/T325 was further selected as the basis for model modification due to its suitability for UHPC systems.

#### 2.3.7. Microscopic Investigation

At the specific curing times, the UHPC sample was crushed into small pieces, which were placed in anhydrous alcohol to stop hydration. Subsequently, the small UHPC piece was transferred into an oven for drying. Upon completion of the drying process, the UHPC piece was ground into powder with a particle size of less than 50 μm. The X-ray diffraction (XRD) analysis of UHPC was conducted using a Rigaku SmartLab SE X-ray diffractometer from Rigaku Corporation, Tokyo, Japan. During testing, a voltage of 40 kV was applied, accompanied by a current of 30 mA. The scanning range and scanning speed were 10–80° (2θ) and of 5°/min, respectively.

In order to better understand the role of coarse aggregates in hydration degree of cementitious materials in the ITZ in UHPC [42], the content of Ca(OH)_2_ was determined by thermogravimetric (TG)/differential thermogravimetric (DTG) thermal analysis. During the test, UHPC samples were heated from 30 °C to 1000 °C at a heating rate of 10 °C/min.

An automatic mercury intrusion porosimetry (MIP) (Auto Pore V 9600) analyzer was used to investigate the pore structure in UHPC (Micromeritics Instrument Corporation, Norcross, GA, USA). In light of the necessity to pull out the admixed steel fiber (with a diameter of 0.2 mm) from the small UHPC piece prior to the MIP analysis, the pore size range during the test was set as 0.005–100 μm.

Aiming at clearly presenting the effect of coarse aggregate on the microstructure of UHPC, the area that contained both coarse aggregate and paste was subjected to scanning electron microscope (SEM) analysis (Hitachi High-Tech Corporation, Tokyo, Japan). During the analysis, the accelerating voltage and beam current were 30 kV and 50 nA, respectively.

## 3. Results and Discussions

### 3.1. Fluidity

**Figure 3:**
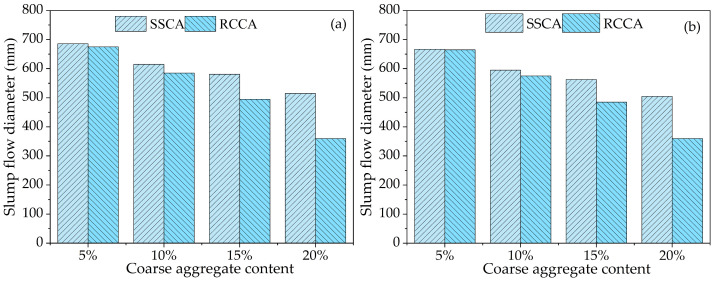
Effect of the different types and contents of coarse aggregate with size range of (**a**) 5–10 mm and (**b**) 5–16 mm on fluidity of UHPC.

Figure 3 illustrates the effect of different types and contents of coarse aggregate on the fluidity of low-carbon UHPC. It is obvious from this figure that with the increase in coarse aggregate content, the fluidity of UHPC decreased, at both 5–10 mm and 5–16 mm size ranges of coarse aggregate. As shown in Figure 3b, UHPC containing 5–16 mm coarse aggregate exhibited a further reduction in fluidity compared with UHPC containing 5–10 mm coarse aggregate. The reduction in fluidity can be attributed not only to the formation of an aggregate skeleton but also to changes in the rheological characteristics of the fresh UHPC. Due to the extremely low water-to-binder ratio and high solid concentration, the workability of UHPC strongly depends on the lubrication effect provided by the cementitious paste. The incorporation of coarse aggregates reduced the volume fraction of free paste available to lubricate solid particles and fibers, resulting in increased interparticle friction and a higher resistance to flow. In addition, larger aggregates promoted the formation of a more stable particle skeleton, which increased the yield stress of the fresh mixture and further restricted the flow of the UHPC matrix.

It is worth noting that among the investigated coarse aggregates, RCCA exhibited the greatest impact on the fluidity of UHPC. This behavior was mainly associated with the porous adhered mortar attached to the surface of RCCA, which absorbed part of the mixing water and reduced the effective water-to-binder ratio of the fresh mixture. Furthermore, the rough surface texture and irregular morphology of RCCA increased particle interlocking and friction resistance, thereby reducing the workability of UHPC [43]. For SSCA, although its surface was relatively denser and smoother than that of RCCA [44], the incorporation of coarse particles still reduces fluidity through aggregate skeleton formation and increased interaction between aggregates and steel fibers [45]. The presence of 2 vol.% steel fibers made the fresh UHPC highly sensitive to aggregate incorporation. As the aggregate content and maximum particle size increased, fiber–aggregate and fiber–fiber interactions became more frequent, hindering particle rearrangement during flow and leading to a further reduction in slump flow. Consequently, the mixture containing 20% SSCA with a particle size of 5–16 mm exhibited a slump flow reduction of 32.21% compared to the reference mix proportion.

### 3.2. Compressive Strength

**Figure 4:**
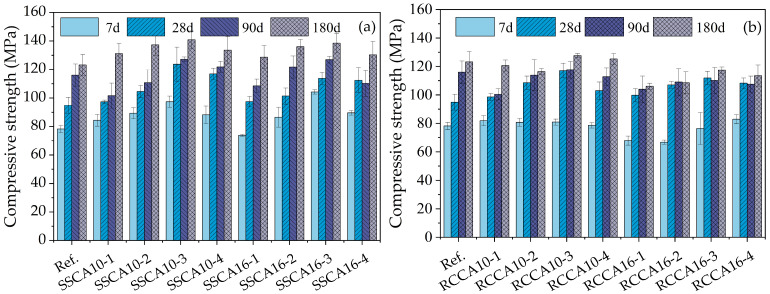
Compressive strength of UHPC containing (**a**) SSCA and (**b**) RCCA at the different curing ages.

Figure 4 shows the compressive strength of UHPC containing two types of coarse aggregates. It can be seen from this figure that all UHPC with or without coarse aggregate exhibited an increase in compressive strength with increasing the curing time. Moreover, when focusing on the effect of the content of coarse aggregate on the compressive strength of UHPC, it normally followed the same pattern of an initial increase followed by a subsequent decline. Notably, the compressive strength reached its maximum value at the content of coarse aggregate being 15%. This is consistent with another study which found that a further increased content of coarse aggregate could lower the mechanical property of UHPC [44,46]. It should be noted that at a replacement level of 15%, the coarse aggregates contributed to a more optimized particle size distribution and reduced the volume of large voids within the composite system, resulting in a denser microstructure and improved load-transfer efficiency.

Specifically, compared to the control UHPC without any coarse aggregate, the compressive strength of mixtures SSCA10-3 and RCCA10-3 that contained 15% SSCA and RCCA with size range of 5–10 mm was improved by 11.89%, 23.32%, and 2.41%, respectively, at the curing age of 28 day. The most significant improvement induced by the addition of SSCA can be due to its robust texture and high compressive strength, as evidenced by the minimal crushing value (Table 2). Furthermore, coarse aggregates can provide a confinement effect on the surrounding cementitious matrix under compressive loading. The presence of rigid aggregate particles restricted the deformation of the matrix and delayed the initiation and propagation of microcracks, thereby enhancing the compressive strength of UHPC. This effect was particularly evident for SSCA due to its high stiffness and low crushing value.

Compared with SSCA, RCCA exhibited only a slight improvement in the compressive strength of UHPC. This is also found in another study [47], which attributed the lack of significant improvement in strength to the brittleness of RA. In this study, the crushing value of RCCA was as high as 24.1%, leading to the lowered compressive strength of UHPC observed in Figure 4b. Although the relatively high crushing value limited the strength enhancement, the porous adhered mortar attached to the aggregate surface may provide a certain internal curing effect. The absorbed water stored within RCCA can be gradually released during hydration, promoting the continued reaction of cementitious materials and contributing to microstructure densification at later ages. In addition, the incorporation of coarse aggregates influenced the development of the ITZ. When the aggregate content was moderate (15%), the beneficial effects associated with improved particle packing, matrix confinement and hydration development outweighed the potential adverse influence of additional interfaces, resulting in the highest compressive strength. However, when the aggregate content further increased to 20%, the number of interfaces and stress concentration sites increased, which may weaken the matrix continuity and lead to a reduction in compressive strength of UHPC.

### 3.3. Internal Relative Humidity

**Figure 5:**
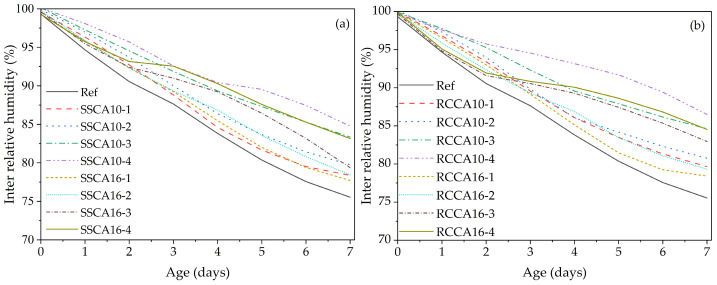
Internal relative humidity of UHPC containing (**a**) SSCA and (**b**) RCCA at the first 7 days.

The internal relative humidity of UHPC at the first 7 days is illustrated in Figure 5. Compared to the control group, the inclusion of coarse aggregate was found to be beneficial in maintaining the internal relative humidity of UHPC. Moreover, as the content of coarse aggregates increased, the internal relative humidity could be maintained at a higher level. This suggests that the addition of coarse aggregate helped keep moisture inside UHPC, preventing it from drying out [48].

At the end of the testing, the final internal relative humidities of mixtures SSCA10-4 and RCCA10-4 containing 20% SA and RA, each with size ranges of 5–10 mm, were recorded at 84.77% and 86.50%, respectively. In contrast, the corresponding values for mixtures SSCA16-4 and RCCA16-4 with the 5–16 mm coarse aggregate were observed to be 78.37%, 83.16% and 84.53%, respectively, as present in Figure 5. In contrast, the ability of RCCA to maintain the internal relative humidity was superior than SSCA. This can be ascribed to the high water absorption rate of RCCA, which was 7.04%, as listed in Table 2. In other words, the water absorbed by RCCA could be released to UHPC matrix when the moisture level inside it dropped [14], contributing to the larger internal relative humidity in mixtures RCCA10-4 and RCCA16-4, as present in Figure 5b.

### 3.4. Autogenous Shrinkage

**Figure 6:**
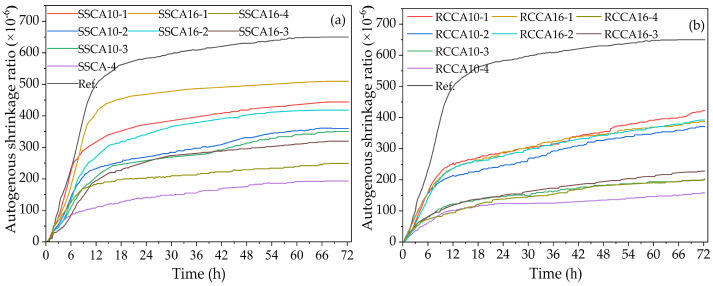
Autogenous shrinkage of UHPC containing (**a**) SSCA and (**b**) RCCA at the first 72 h.

The autogenous shrinkage of UHPC at the first 72 h is illustrated in Figure 6. It is obvious from this figure that the addition of coarse aggregates could effectively reduce the autogenous shrinkage of UHPC. Furthermore, the shrinkage curve of the control UHPC exhibited a plateau following a time of 18 h, whereas the shrinkage curve of UHPC incorporating coarse aggregate generally began to stabilize after approximately 12 h. In other words, the employment of coarse aggregate accelerated the termination of autogenous shrinkage of UHPC.

After 72 h, the final autogenous shrinkage values of mixtures SSCA10-4 and RCCA10-4 were 193 με, and 158 με (Figure 6), respectively, which were reduced by 70.31%, and 75.69% compared to that of the control UHPC. It should be noted that among the employed two types of coarse aggregate, RCCA was the most effective one in reducing the autogenous shrinkage of UHPC. This can be explained by the aforementioned higher water absorption capacity of RCCA, which could facilitate the release of the absorbed moisture into the surrounding matrix, ensuring the stability of the relative humidity inside UHPC and thus mitigating the autogenous shrinkage [9,19,49]. Moreover, at the same content level, coarse aggregate with size range of 5–10 mm exhibited a greater efficacy in reducing the autogenous shrinkage of UHPC compared to that with the size ranging from 5 mm to 16 mm. It can be concluded that small-sized coarse aggregate featured a superior capacity for mitigating autogenous shrinkage than its larger counterpart.

### 3.5. Drying Shrinkage

**Figure 7:**
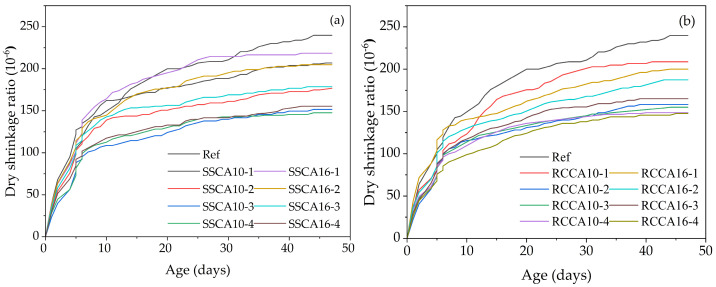
Drying shrinkage of UHPC containing (**a**) SSCA and (**b**) RCCA until the age of 45 days.

Similarly with the autogenous shrinkage, the inclusion of coarse aggregate can also reduce the drying shrinkage of UHPC, as depicted in Figure 7. Moreover, as the content of coarse aggregate increased, the reduction effect in drying shrinkage became more significant. This implies that the application of coarse aggregate was an effective approach to mitigate the drying shrinkage and delay the initiation of cracking of UHPC [21].

At the end of testing (45 days), the final drying shrinkages of mixtures SSCA10-4 and RCCA10-4 were 147.6 με, and 148.5 με, respectively, which were reduced by 38.06%, and 37.73% compared to UHPC without coarse aggregate. It can be concluded that SSCA and RCCA played a more important role in mitigating the drying shrinkage of UHPC. This phenomenon can be attributed to the porous nature of RCCA and SSCA, both of which can absorb water during the preparation of UHPC. When UHPC containing RCCA or SSCA was exposed to a drying environment, the moisture retained in aggregates can be gradually released to the UHPC matrix, thereby inhibiting the development of shrinkage.

### 3.6. Shrinkage Prediction Model

#### 3.6.1. Evaluation of Existing Models

**Figure 8:**
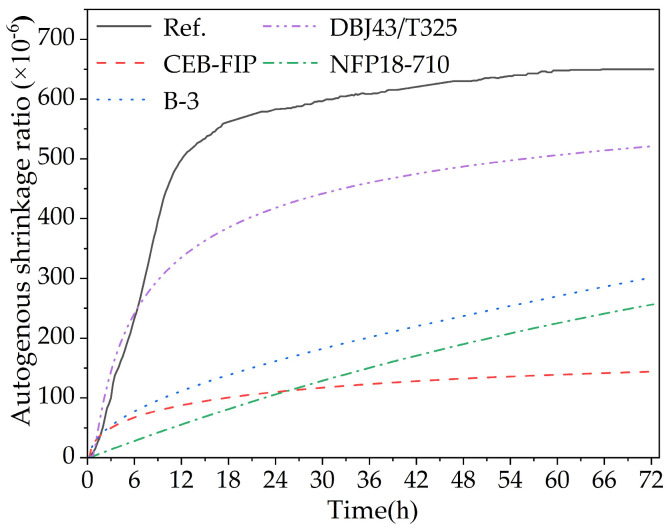
Comparison in shrinkage of UHPC between the measured value and the predicted values from different models.

The theoretical shrinkage rates at different ages, calculated using the four shrinkage strain prediction models (CEB-FIP model, JTG D62-2004 model, NF P18-710-2016 model, and DBJ 43/T325-2017 model), were compared with the measured shrinkage rates of UHPC without coarse aggregates. The results for the shrinkage curves are shown in Figure 8 and Table 4. As can be seen from Table 4 and Figure 8, there is a significant discrepancy between the measured shrinkage strain values of UHPC at different ages and the values calculated by the theoretical models. The ranking of calculation accuracy is as follows: DBJ43/T325 model > B3 model > NF P18-710 model > CEB-FIP model. The ratios indicate that all four models underestimate the autogenous shrinkage of UHPC, with the CEB-FIP model exhibiting the largest error. This is because the CEB-FIP model is based on normal concrete and is not suitable for UHPC without coarse aggregates. The DBJ43/T325 model demonstrates the highest accuracy, and its shrinkage curve aligns well with the trend of the measured data.

#### 3.6.2. Modified DBJ43/T235 Model

Given the highest accuracy of the DBJ43/T325 model, it was modified by considering the influence of the type and content of coarse aggregate. The modified DBJ43/T325 model is shown in Equation (1).

(1) $$\gamma_{RH}\varepsilon_{sh}=\gamma_{t}\times 700e^{-\left(\frac{2.5}{\sqrt{t-0.5}}\right)}+\gamma_{c}$$

where $\gamma_{t}$ is the influence coefficient of the coarse aggregate type on shrinkage and $\gamma_{c}$ is the influence coefficient of the coarse aggregate content on shrinkage.

**Figure 9:**
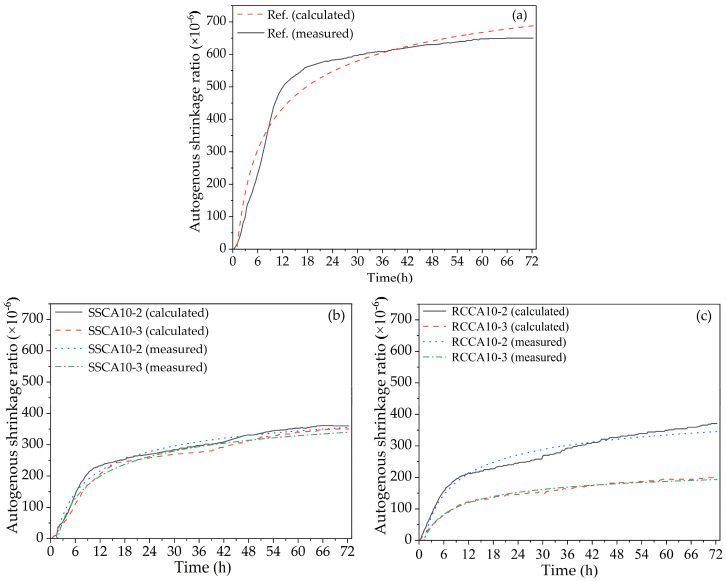
The calculated and measured autogenous shrinkage of UHPC containing (**a**) no coarse aggregate, (**b**) SSCA and (**c**) RCCA.

The actual shrinkage rates of UHPC containing 10% and 15% of SCCA and RCCA, respectively, were fitted using the model. The fitting results are shown in Figure 9. The figure indicates that for the reference group without coarse aggregate, the $\gamma_{t}$ coefficient is zero, and consequently, the model’s accuracy for this group is significantly lower than for the experimental groups containing coarse aggregate, where the $\gamma_{t}$ coefficient was applied. Furthermore, it can be clearly observed that the trend of the model-predicted curves aligns with that of the measured data curves, with differences appearing only in the final shrinkage values for some experimental groups.

After multiple iterations, the shrinkage influence coefficients for different types of coarse aggregates and various content levels were obtained, as shown in Table 5. The table indicates that the goodness-of-fit ($R^{2}$) for the baseline group without coarse aggregate is 0.94, while the $R^{2}$ values for curves under different types and content levels of coarse aggregate all exceed 0.97. This demonstrates that the modified DBJ43/T325 prediction model exhibits a good agreement with the actual shrinkage rates of UHPC containing coarse aggregate. Compared to the unmodified DBJ43/T325 function, the improved model more accurately reflects the autogenous shrinkage behavior of UHPC with the inclusion of coarse aggregates.

### 3.7. XRD Analysis

**Figure 10:**
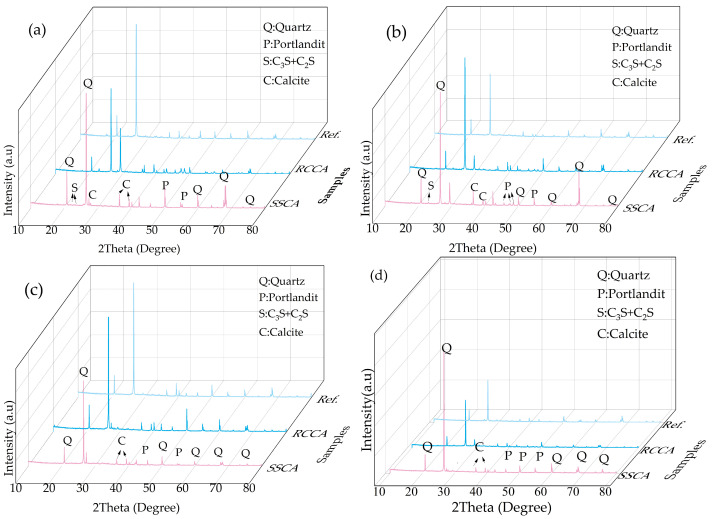
XRD patterns of UHPC at curing time of (**a**) 7 days, (**b**) 28 days, (**c**) 90 days and (**d**) 180 days.

Figure 10 shows XRD patterns of UHPC with or without coarse aggregate at different curing times. It can be seen from this figure the peak corresponding to cement clinker phases C_3_S and C_2_S decreased with increasing the curing time. This is due to the cement hydration, where C_3_S and C_2_S were consumed to generate hydration products, including calcium silicate hydrate (C-S-H) gel and Ca(OH)_2_. At the same time, Ca(OH)_2_ can react with active silica and alumina in SCM to form additional hydration product, calcium aluminosilicate hydrate (C-A-S-H) gel, as evidenced by the gradually declining peak of Portlandite in Figure 10. Both the cement hydration and pozzolanic reaction of SCM would contribute to the development of the mechanical property of UHPC with the increase in curing time. It should be noted that the XRD analysis presented herein is primarily qualitative. Therefore, the diffraction peak intensities were used to indicate the relative evolution of hydration products rather than provide quantitative phase contents. To further verify the hydration evolution and Ca(OH)_2_ consumption, the XRD results were interpreted together with the TG/DTG analysis presented later.

In Figure 10a, the diffraction peak corresponding to Ca(OH)_2_ at 50.3° in UHPC containing RCCA was more pronounced than that in UHPC with SSCA. This observation suggests that the addition of RCCA increased the formation of Ca(OH)_2_ at an early age (7 days), which can facilitate the pozzolanic reaction of fly ash in the following curing ages. However, as the hydration of cement and the pozzolanic reaction of SCM proceeded, the strength of UHPC matrix developed, ultimately surpassing that of RCCA itself. In this case, the initial failure would occur in RCCA, deteriorating the development of strength of UHPC. Consequently, the effect of RCCA on the later-age strength of UHPC was relatively small, as evidenced by the lowered peak of Ca(OH)_2_ in Figure 10c,d. This is consistent with the development of compressive strength.

### 3.8. TG/DTG Analysis

**Figure 11:**
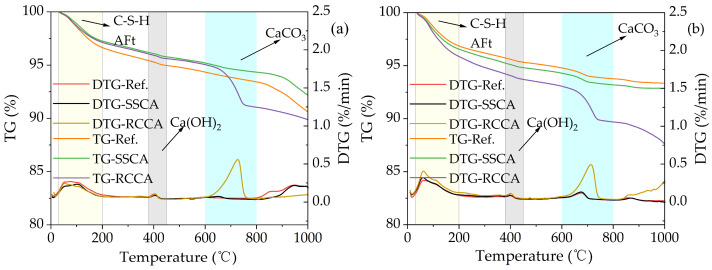
TG and DTG curves of UHPC at the curing time of (**a**) 28 days and (**b**) 180 days.

Figure 11 presents the TG/DTG curves of the ITZ in the control UHPC without any coarse aggregate and UHPC containing 20% coarse aggregate with the size range of 5–10 mm. It can be observed from this figure that UHPC at different ages exhibited a significant mass loss in the temperature range of 30–200 °C. This is mainly due to the dehydration of physically bound water in C-S-H gel and ettringite (AFt). Moreover, the peak values observed in DTG curves in this range exhibited an increasing trend when increasing the curing time from 28 days to 180 days, as depicted in Figure 11b. This trend indicates an increase in the content of bound water, which in turn suggests a higher content of C-S-H and AFt phases. At the macroscopic level, it can be reflected by the enhancement in compressive strength of UHPC with the curing time.

In the temperature range around 400 °C, the mass loss of UHPC corresponds to the decomposition of Ca(OH)_2_. In this case, the content of Ca(OH)_2_ can be calculated using the TG data. Table 6 shows the obtained content of Ca(OH)_2_ in the ITZ of UHPC at different ages. Interestingly, the evolution of Ca(OH)_2_ observed in the ITZ differed from that identified in the bulk matrix by XRD analysis. This apparent difference may be attributed to the heterogeneous distribution of hydration products within UHPC. Previous studies have reported that Portlandite tends to accumulate preferentially in the ITZ due to wall effects and local hydration heterogeneity, particularly in recycled aggregate systems [50]. Meanwhile, in UHPC containing SCMs, Ca(OH)_2_ presented in the bulk matrix can be progressively consumed through pozzolanic reactions, resulting in the formation of additional C-S-H gel [51]. Therefore, a higher Ca(OH)_2_ content in the ITZ did not necessarily contradict the lower Portlandite intensity observed in the matrix by XRD analysis. The relatively high Ca(OH)_2_ content detected in the ITZ of RCCA-containing UHPC, together with the porous adhered mortar surrounding recycled aggregates, may contribute to the formation of a relatively weaker ITZ [52]. This phenomenon could partially explain the comparatively limited strength enhancement observed in UHPC containing RCCA.

### 3.9. Pore Structure

**Figure 12:**
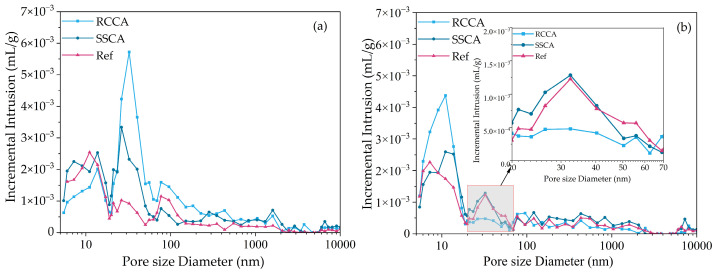
MIP analysis of UHPC at the curing ages of (**a**) 7 days and (**b**) 28 days.

Figure 12 presents the incremental intrusion curves of mercury during the MIP analysis. It is well known that the critical pore radius corresponds to the peak in the curve, which means the aperture of pore that exhibits the highest abundance within the detected range. It can be seen from this figure that the critical pore size of UHPC decreased when increasing the curing time from 7 days to 28 days. Specifically, at the curing time of 7 days, UHPC incorporating RCCA and SSCA featured critical pore sizes of 32.4 nm and 26.3 nm, respectively. In contrast, after 28 days of curing, these values diminished significantly to 11.05 nm and 11.04 nm, respectively. This indicates that the formation of hydration products can refine the pore structure.

Table 7 shows the pore structure parameters of UHPC with or without coarse aggregate. The data in this table shows that the porosity of UHPC was very low. It should be noted that UHPC containing SSCA and RCCA featured a higher porosity than the control UHPC. This is mainly due to the inherent porous nature of the coarse aggregates, which absorbed water from the UHPC matrix during the early age. This absorption can facilitate the water transport in the matrix, which contributed to the formation of transport channels and thus the increased porosity. However, with increasing the age, the moisture inside the coarse aggregate can be released to the UHPC matrix, participating in the cement hydration and pozzolanic reaction of SCM. Consequently, the generation of hydration products can lead to a reduction in porosity, as detailed in the 28-day porosity in Table 7. The formation of additional hydration products can also refine the pore structure in UHPC, especially for mixture RCCA16-4, as evidenced by the significant reduction in the content of pores in the range of 20–100 nm from 7 days to 28 days, as present in Table 7.

It should be noted that an increase in total porosity did not necessarily contradict the observed pore refinement. Porosity reflected the overall volume of voids within the materials, whereas the pore size distribution described how these voids were distributed across different size ranges. The incorporation of RCCA and SSCA may introduce additional pores associated with the aggregates and the ITZ, resulting in a slightly higher total porosity. However, the internal curing effect provided by these aggregates promoted continued hydration and pozzolanic reactions, leading to the conversion of large capillary pores into finer gel pores. Consequently, although the total porosity may remain slightly higher than that of the control mixture, the critical pore size and the proportion of harmful pores were significantly reduced. This refinement of the pore structure was generally considered beneficial for mechanical performance and durability because transport properties were more strongly influenced by pore connectivity and pore size distribution than by total porosity alone.

### 3.10. Microstructure

**Figure 13:**
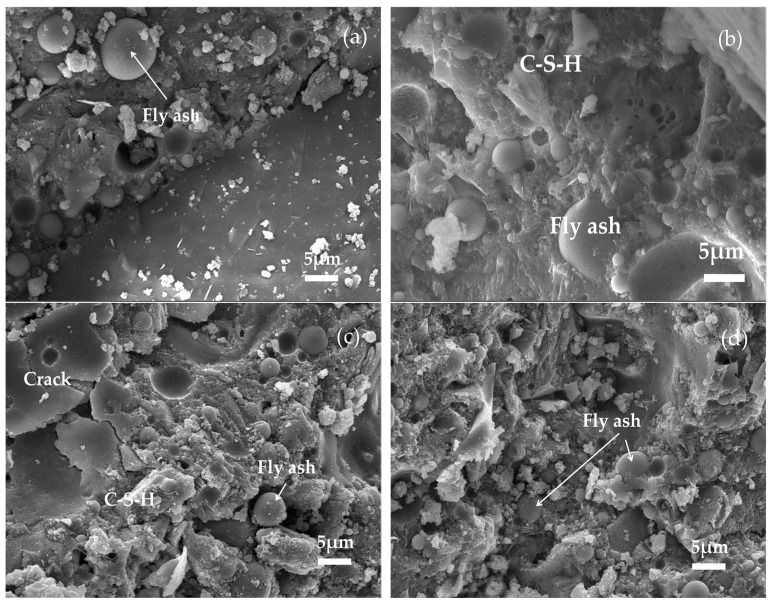
SEM images of the control UHPC at different ages of (**a**) 7 days, (**b**) 28 days, (**c**) 90 days and (**d**) 180 days.

Figure 13 shows the SEM images of the control UHPC without any coarse aggregate at different curing ages. At the age of 7 days (Figure 13a), it is obvious that there existed many unreacted fly ash particles. In addition, one can also observe the presence of C-S-H gels, hexagonal structured Ca(OH)_2_ crystals, and acicular ettringite. As curing time increased, the content of spherical fly ash particles gradually decreased, accompanying with the formation of more hydration products, as depicted in Figure 13c,d. This indicates that the pozzolanic reaction of SCM played a more important role in densifying the microstructure of UHPC at later ages.

**Figure 14:**
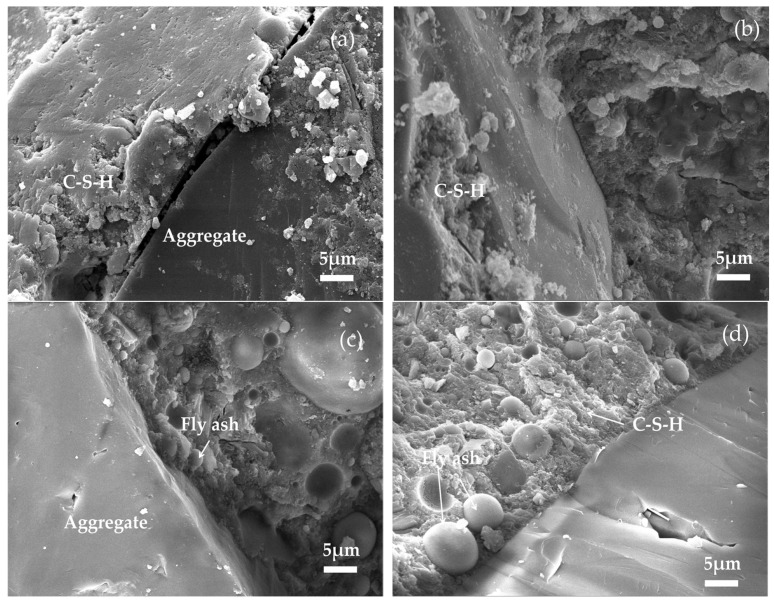
SEM images of UHPC containing SSCA at different ages of (**a**) 7 days, (**b**) 28 days, (**c**) 90 days and (**d**) 180 days.

At the curing time of 7 days, an obvious crack appeared along the coarse aggregate boundary. However, the microstructure of the ITZ became densified with increasing the curing time, as illustrated in Figure 14b–d. This can be primarily attributed to the water absorption and subsequent re-release of moisture from SSCA, which played an important role in facilitating hydration in the UHPC matrix.

**Figure 15:**
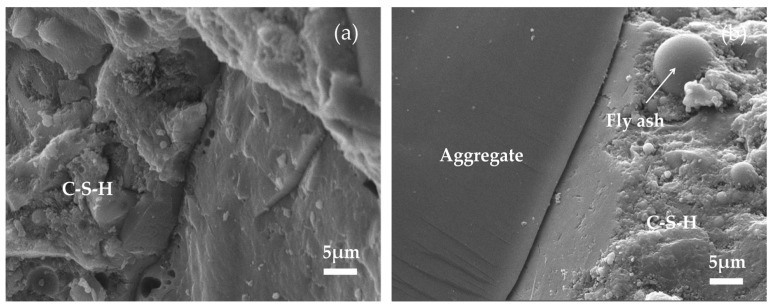
SEM images of (**a**) new ITZ and (**b**) old ITZ in UHPC containing RCCA at the curing time of 7 days.

In light of the simultaneous presence of ITZs between RA and old mortar, as well as between RCCA and new UHPC matrix in UHPC containing RCCA, it is necessary to conduct a comparative analysis of these two distinct types of ITZ. Figure 15 shows the morphology of the new and old ITZs in RCCA-contained UHPC at the curing time of 7 days. It can be seen from this figure that a large amount of Ca(OH)_2_ was present in the new ITZ, while a crack can be observed in the old ITZ. Normally, the hexagonal crystals of Ca(OH)_2_ can concentrate at the interface between coarse aggregate and UHPC matrix due to the wall effect and localized heterogeneity of hydration products [53]. However, the morphology of Ca(OH)_2_ usually subjected to alteration as a result of various factors, including the growth space, hydration temperature, and the presence of impurities in the system, making the accurate identification of Ca(OH)_2_ difficult in SEM observation [54], as illustrated in Figure 15a. At the same time, it can be observed that the surface of fly ash particles was no longer smooth, indicating that pozzolanic reactions had already occurred at this stage.

**Figure 16:**
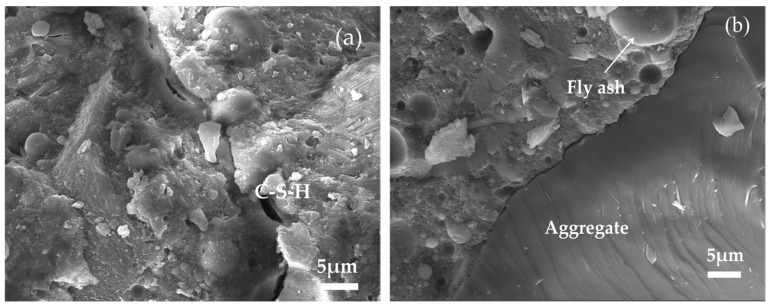
SEM images of (**a**) new ITZ and (**b**) old ITZ in UHPC containing RCCA at the curing time of 90 days.

Figure 16 presents the comparison of the new and old ITZs in UHPC containing RCCA at the curing time of 90 days. At this time, more hydration products were generated in the new ITZ, resulting in a denser microstructure. This observation was consistent with the TG results, which showed an increase in chemically bound water, and with the MIP results, which indicated a refinement of the pore structure with curing age. Similar densification of newly formed ITZs had also been reported in recycled aggregate concrete systems [55].

Meanwhile, there still existed a clear interface between coarse aggregate and the old mortar. In addition, visible microcracks could still be observed in the old ITZ. Previous studies have reported that the old ITZ associated with adhered mortar generally possessed higher porosity, a higher concentration of microcracks, and weaker mechanical properties than newly formed ITZs [56]. Therefore, although the new ITZ continued to be strengthened through ongoing hydration and pozzolanic reactions, the old ITZ may remain the mechanically weaker region in RCCA-containing UHPC. This phenomenon could partially explain why RCCA exhibited only a limited contribution to the later-age strength development of UHPC.

### 3.11. Engineering Applicability and Durability Implications

The findings of this study demonstrated the practical feasibility of incorporating SSCA and RCCA into low-carbon UHPC containing high volumes of SCMs. From an engineering perspective, the significant reduction in autogenous and drying shrinkage achieved through the incorporation of coarse aggregates can effectively mitigate the risk of early-age cracking, which is particularly beneficial for bridge decks, precast components, repair materials, and thin-walled structural elements where dimensional stability is critical. In addition, the water absorption and subsequent moisture release of SSCA and RCCA contributed to an internal curing effect, maintaining a higher internal relative humidity and promoting continued hydration. Microstructural analyses further revealed pore structure refinement and progressive densification of the ITZ, indicating potential benefits for long-term durability. Although the incorporation of coarse aggregates resulted in a slight increase in total porosity, the reduction in critical pore size may help limit the ingress of aggressive substances, such as chlorides and sulfates, thereby contributing to enhanced durability and service life. Nevertheless, the durability implications discussed in this study are primarily inferred from microstructural observations on chloride penetration resistance, carbonation resistance, freeze–thaw durability, and long-term field performance are required to comprehensively evaluate the engineering applicability of low-carbon UHPC incorporating SSCA and RCCA.

## 4. Conclusions

This study investigated the effects of coarse aggregate type, content and particle size on the mechanical performance, internal humidity evolution, shrinkage behavior and microstructure of low-carbon UHPC incorporating a high volume of supplementary cementitious materials and aeolian sand. Based on the experimental results, the following conclusions can be drawn.

(1) The incorporation of coarse aggregate modified the fresh-state behavior of UHPC by reducing its fluidity, mainly owing to aggregate skeleton formation, increased interparticle friction and water absorption. The reduction became more pronounced with increasing aggregate size, while RCCA caused the greatest loss of workability because of its relatively high water absorption capacity.
(2) Coarse aggregate effectively enhanced the compressive strength of low-carbon UHPC when an appropriate replacement level was adopted. An optimum aggregate content of 15% was identified in this study, at which the 28-day compressive strength increased by up to 23.32% compared with the control mixture. The improvement was attributed to enhanced particles packing, aggregate restraint effects, and continued hydration promoted by internal curing.
(3) Steel slag aggregate and recycled concrete coarse aggregate acted as internal curing water reservoirs, mitigating self-desiccation and maintaining a higher internal relative humidity. Consequently, both autogenous shrinkage and drying shrinkage were significantly reduced. The greatest shrinkage reduction was achieved using 20% RCCA with a particle size of 5–10 mm, resulting in decreases of 75.69% and 62.67% in autogenous and drying shrinkage, respectively.
(4) Microstructural analyses demonstrated that the incorporation of coarse aggregate altered the hydration environment and pore structure of UHPC. Although a slight increase in total porosity was observed, the critical pore size was substantially reduced, indicating pore structure refinement. XRD, TG/DTG, MIP and SEM results collectively confirmed that ongoing hydration and pozzolanic reactions promoted microstructural densification, particularly in the newly formed ITZ surrounding coarse aggregate.
(5) The combined use of coarse aggregate and high-volume SCMs provided a feasible strategy for producing low-carbon UHPC with improved volume stability while maintaining excellent mechanical performance. However, the present study focused primarily on shrinkage and compressive strength under laboratory conditions. Further investigations on long-term durability, transport properties, cracking resistance and field-scale application are required before practical implementation.

## Figures and Tables

**Table 1:** Chemical composition of binders (wt.%).

Chemical Composition	Cement	Fly Ash	GGBS	Silica Fume
SiO_2_	21.50	66.67	32.08	94.56
CaO	59.81	3.05	38.09	0.14
Al_2_O_3_	2.06	18.97	15.06	0.09
SO_3_	5.86	0.30	8.26	1.25
K_2_O	2.85	4.39	1.93	0.54
Na_2_O	0.20	3.05	0.34	0.29
P_2_O_5_	0.67	0.30	0.47	0.14
LOI *	3.70	3.10	0.17	2.62

* LOI: Loss on ignition.

**Table 2:** Physical properties of coarse aggregates.

Aggregate	Crushing Value (%)	Water Absorption (%)	Apparent Density (kg/m^3^)
SSCA	5.61	3.54	3130
RCCA	24.10	7.04	2630

**Table 3:** Mix design of UHPC.

Group	UHPC Matrix (vol.%)	Coarse Aggregate (vol.%)	
		5–10 mm	5–16 mm
Ref.	100	0	0
SSCA/RCCA 10-1	95	5	0
SSCA/RCCA 10-2	90	10	0
SSCA/RCCA 10-3	85	15	0
SSCA/RCCA 10-4	80	20	0
SSCA/RCCA 16-1	95	0	5
SSCA/RCCA 16-2	90	0	10
SSCA/RCCA 16-3	85	0	15
SSCA/RCCA 16-4	80	0	20

**Table 4:** Comparison between measured values and model calculated values of UHPC.

Time/h	$\varepsilon_{\mathrm{Ref}}/\varepsilon_{\mathrm{CEB}\text{-}\mathrm{FIP}}$	$\varepsilon_{\mathrm{Ref}}/\varepsilon_{B\text{-}3}$	$\varepsilon_{\mathrm{Ref}}/\varepsilon_{\mathrm{NFP}18\text{-}710}$	$\varepsilon_{\mathrm{Ref}}/\varepsilon_{\mathrm{DBJ}43/T325}$
12 h	5.69	4.47	8.99	1.49
24 h	5.31	3.61	5.52	1.39
36 h	4.94	3.02	4.05	1.32
48 h	4.77	2.66	3.32	1.29
72 h	4.51	2.16	2.54	1.25
Average	5.04	3.18	4.88	1.35
Coefficient of Variation	9.20%	28.06%	52.14%	7.00%

**Table 5:** Coarse aggregate influence coefficients of autogenous shrinkage of UHPC.

Coarse Aggregate Type	Reference	SSCA		RCCA	
Coarse Aggregate Content	0%	10%	15%	10%	15%
$\gamma_{t}$	1.3134	0.7444	0.7533	0.7170	0.3945
$\gamma_{c}$	0	−32.87	52.80	−28.25	−12.28
$R^{2}$	0.9446	0.9822	0.9777	0.9575	0.9852

**Table 6:** The content of Ca(OH)_2_ in the ITZ of UHPC obtained using the TG analysis.

Ca(OH)_2_ Content (%)	Ref	SSCA	RCCA
28d	1.476	1.362	1.480
180d	1.622	1.490	1.912

**Table 7:** Pore structure parameters of UHPC at different ages.

Mix	Curing Time (Day)	Porosity (%)	Pore Size Distribution (%)		
			<20 nm	20–100 nm	>100 nm
Ref.	7	7.3	19.5	35.8	44.7
	28	6.9	34.3	22.5	43.2
SSCA16-4	7	10.9	30.1	22.7	47.2
	28	8.9	33.5	20.9	45.5
RCCA16-4	7	11.9	16.2	43.3	40.5
	28	7.6	52.5	11.6	35.9

## Data Availability

The original contributions presented in this study are included in the article. Further inquiries can be directed to the corresponding authors.
